# Innovation in healthcare: leadership perceptions about the innovation characteristics of artificial intelligence—a qualitative interview study with healthcare leaders in Sweden

**DOI:** 10.1186/s43058-023-00458-8

**Published:** 2023-07-18

**Authors:** Margit Neher, Lena Petersson, Jens M Nygren, Petra Svedberg, Ingrid Larsson, Per Nilsen

**Affiliations:** 1 School of Health and Welfare, Halmstad University, Box 823, SE-30118 Halmstad, Sweden; 2 Department of Health, Medicine and Caring Sciences, Division of Public Health, Faculty of Health Sciences, Linköping University, Linköping, Sweden

**Keywords:** Artificial intelligence, Healthcare, Implementation, Healthcare leaders, Organisational change, Qualitative methods, Stakeholders, Consolidated framework of implementation research

## Abstract

**Background:**

Despite the extensive hopes and expectations for value creation resulting from the implementation of artificial intelligence (AI) applications in healthcare, research has predominantly been technology-centric rather than focused on the many changes that are required in clinical practice for the technology to be successfully implemented. The importance of leaders in the successful implementation of innovations in healthcare is well recognised, yet their perspectives on the specific innovation characteristics of AI are still unknown. The aim of this study was therefore to explore the perceptions of leaders in healthcare concerning the innovation characteristics of AI intended to be implemented into their organisation.

**Methods:**

The study had a deductive qualitative design, using constructs from the innovation domain in the Consolidated Framework for Implementation Research (CFIR). Interviews were conducted with 26 leaders in healthcare.

**Results:**

Participants perceived that AI could provide relative advantages when it came to care management, supporting clinical decisions, and the early detection of disease and risk of disease. The development of AI in the organisation itself was perceived as the main current innovation source. The evidence base behind AI technology was questioned, in relation to its transparency, potential quality improvement, and safety risks. Although the participants acknowledged AI to be superior to human action in terms of effectiveness and precision in some situations, they also expressed uncertainty about the adaptability and trialability of AI. Complexities such as the characteristics of the technology, the lack of conceptual consensus about AI, and the need for a variety of implementation strategies to accomplish transformative change in practice were identified, as were uncertainties about the costs involved in AI implementation.

**Conclusion:**

Healthcare leaders not only saw potential in the technology and its use in practice, but also felt that AI’s opacity limits its evidence strength and that complexities in relation to AI itself and its implementation influence its current use in healthcare practice. More research is needed based on actual experiences using AI applications in real-world situations and their impact on clinical practice. New theories, models, and frameworks may need to be developed to meet challenges related to the implementation of AI in healthcare.

**Supplementary Information:**

The online version contains supplementary material available at 10.1186/s43058-023-00458-8.

Contributions to the literature • The findings reflect perceptions by technically and clinically experienced leaders that may challenge some of the prevalent views concerning the innovative characteristics of AI in healthcare.

• Leaders in healthcare are under-researched as a stakeholder group and of key importance for successful implementation of AI in healthcare.

• The findings probe deeper into the understanding of how the perception of leaders in healthcare on the main characteristics of AI in healthcare aligns with current theory on characteristics within the “innovation domain” as described in CFIR.

• The study contributes to Implementation Science knowledge by deductive mapping of empirical data to one of the CFIR framework domains, to further develop the understanding of the domain and its relevance in the implementation of AI in healthcare.

## Background

Artificial intelligence (AI) has the potential to enhance healthcare in many high-income countries through improved efficiency, quality, and clinical and health outcomes [1, 2]. However, there are substantial challenges to the implementation of AI-based applications in healthcare, as for many other types of innovations in healthcare. AI can be defined as “a machine-based system that can, for a given set of human-defined objectives, make predictions, recommendations, or decisions influencing real or virtual environments … with varying levels of autonomy” [3, 4]. The technology behind AI applications is developing at breakneck speed and is pushing the boundaries of what is technologically possible, introducing expectations for accomplishing improvements in cost-effectiveness, staff workflow, and patient empowerment. To achieve these results, the European Union highlights the importance of safely introducing AI into healthcare, and for the creation and adoption of regulatory frameworks based on human rights and fundamental values [5]. However, while interest in AI is on the rise and the number of explorative and proof-of-concept projects are growing, the level of diffusion is still relatively low in healthcare [2, 6, 7]. There are several potential barriers to using AI in healthcare related to data-based, methodological, technological, regulatory, policy, and human factors [8] and evidence to support implementation strategies to tackle these barriers is still at an early stage [7]. The Consolidated Framework for Implementation Research (CFIR) describes and predicts barriers and facilitators for implementation connected to the characteristics of five theory domains: innovation, outer setting, inner setting, individuals and implementation processes. The degree to which different characteristics of the innovation itself are perceived as appropriate, acceptable, and feasible by individuals involved in the implementation process is key for facilitating the adoption of an innovation [9]. Perceptions of innovation characteristics have been found to be relevant for the implementation of many types of innovations across diverse settings, but how they influence the implementation of AI remains largely unexplored [1, 10, 11].

Implementation science highlights the importance of accounting for stakeholder views in the initial phase of the implementation, as it is key to knowing how different stakeholder groups perceive the characteristics of an innovation and its potential impact on population health and its value for patients [12]. For successful implementation, it is especially important to understand and consider the perspective of leaders [13] yet this is one of the stakeholder groups that has received relatively little attention in research regarding AI implementation [14]. Leaders are usually important in promoting organisational capacity to initiate, guide, and accomplish implementation processes [15–18]. Still, research on leadership in implementation science is still underdeveloped, particularly in relation to the implementation of AI in healthcare, where the main focus has been on other stakeholder groups like healthcare professionals [19–21], patients [22], and industry partners [23]. The paucity of knowledge concerning the importance of leadership in the successful implementation of AI in healthcare has been illustrated in two recent scoping reviews which highlighted that few studies mentioned leadership in relation to AI implementation [24] and that there is a need for more theory-based research on the implementation of AI [7, 25].

Considering the importance of leadership for the implementation, adoption, and use of AI, this study addresses a significant knowledge gap: it aims to explore the perceptions of healthcare leaders in clinical and administrative roles concerning the innovation characteristics of AI as an innovation to be implemented into their organisation.

## Methods

This study was conducted within the frame of a research program “Toward Successful Implementation of Artificial Intelligence in Health Care Practice” [6] with the objective of developing a theoretically and empirically informed framework for AI implementation in healthcare that can be used to facilitate AI implementation in routine healthcare practice. Some of the research activities in this program include mapping different stakeholder perspectives, including those of leaders, healthcare professionals, and patients, with the purpose of understanding contextual aspects regarding requirements, barriers, and enabling factors for the implementation of AI systems in practice. The current study is an important contribution to research on AI implementation in healthcare, offering insights into healthcare leader’s perspectives on the innovative characteristics of AI.

### Design

The study has a qualitative design and adopts a directed approach to qualitative content analysis [26] meaning that the structure of the deductive data analysis was operationalised on the basis of previous knowledge, models, or theory. The Consolidated Framework for Implementation Research (CFIR) outlines determinants that influence implementation efforts, and provides concepts, terms, and definitions to aid researchers in navigating the complex research and practice field. Constructs from the innovation domain in CFIR are used as a deductive tool in the data analysis. The study is reported in accordance with the Consolidated Criteria for Reporting Qualitative Research 32-item checklist to ensure trustworthiness (Additional file 1) [27].

### Theoretical framework

In this study, the CFIR framework was used as a theoretical foundation. The CFIR is intended to collect data from individuals who have power and influence over implementation outcomes [9] and was judged to be appropriate to guide our analysis. In this study, we limited the analysis to the framework’s innovation domain. CFIR’s innovation domain includes eight constructs (innovation source, evidence base, relative advantage, trialability, adaptability, complexity, design, and cost) (Table 1).

**Table 1:** Definitions of the constructs from the innovation domain in the updated Consolidated Framework for Implementation Research (CFIR) [9]. The construct definitions are adapted to AI as an innovation to be adopted into the organisation

**Innovation domain**	
**Construct name**	**Construct definition** *The degree to which:*
Innovation source	The group that developed and/or visibly sponsored use of AI is reputable, credible, and/or trustworthy
Innovation evidence base	The AI innovation has robust evidence supporting its effectiveness
Innovation relative advantage	The AI innovation is better than other available innovations or current practice
Innovation adaptability	The AI innovation can be modified, tailored, or refined to fit local context or needs
Innovation trialability	The AI innovation can be tested or piloted on a small scale and can be retracted if necessary
Innovation complexity	The AI innovation is complicated; which may be reflected by its scope and/or the nature and number of the connections and steps involved in the process
Innovation design	The AI innovation is well-designed and well-packaged, including how it is assembled, bundled, and presented
Innovation cost	The AI innovation purchase and operating costs are affordable

### Setting

The study was conducted in southwest Sweden. Participants were employed in a county council that, over the last decade, has invested financial, personnel, and service resources to develop capacity to structure and analyse healthcare data to generate information on which improvement initiatives can be based [28]. The deployment of AI in the organisation is at an early stage.

### Participants and recruitment

The purposeful recruitment of study participants from the county council focused on leaders in a position to potentially influence the implementation of AI. The study participants belong to a group of high-level county council managers with ‘working knowledge’ related both to AI and to implementing innovations. They had an understanding of the members in the setting and its contextual influences. Damschroder et al. (2022) refer to these individuals as “individuals with a high level of authority, including key decision-makers, executive leaders, or directors” [9].

The number of interviewees was consequently increased organically through a snowballing technique until a sense of the multiple types of leaders emerged and no further roles in the representation of leadership functions within the county council’s organisation were identified. A total of 28 participants were invited to participate in the study via an email including study information. Of these, 26 individuals (18 men and 8 women) were willing to participate in interviews.

Fourteen participants worked in top-level management functions in healthcare. Two participants were top-level regional politicians. Two participants had a technical advisory function. Eight participants had a quality development portfolio in their remit and worked in a strategic role either at an intermediate level or in local quality development roles. Two participants worked in primary healthcare and two worked in secondary care.

### Data collection

The interviews were conducted via phone or video communication by a female researcher (LP, trained in work technology environments, PhD) and a male colleague (DT, trained in management research, PhD); neither of whom had any previous relationship with the participants. The interviews were conducted with each participant on a one-to-one basis on one occasion. A semi-structured approach was used, and the participants were asked to share their perspectives of AI as a phenomenon, their experiences with AI, and their perceptions of what could hinder or facilitate implementation of AI in their workplace (Additional file 2). Although AI systems are designed to operate with varying levels of autonomy, the interview questions did not differentiate between different kinds of AI technology. Two pilot interviews were conducted to test the questions, and since no adjustments were required, these interviews were included in the study. The interviews took place between October 2020 and May 2021 and lasted between 30 and 120 min, with a total length of 23 h and 49 min. The interviews were audio-recorded and transcribed verbatim.

### Data analysis

The study used qualitative content analysis with a directed (deductive) approach, using a stepwise method described by Hsieh and Shannon [25]. The analysis of the qualitative data was undertaken with the software program Nvivo (NVivo 14 for Windows). In the first step of the analysis, all transcripts were read in their entirety by all authors. In the second step, PN and MN constructed a codebook (Table 1) based on the definitions of the eight constructs in CFIR’s innovation domain [9] and adapted these to the study context by operationalising them to fit the innovation (AI). While the definitions adhered closely to the original content of the constructs, the adapted definitions were designed to serve as codes and facilitate the qualitative analysis of the data. The definitions were iteratively discussed and ultimately finalised by the authors. The codes based on these constructs formed the main categories. In the third step, the codes were applied to each interview. The first author (MN) allocated meaning units to the main categories, using the descriptions for each construct in the codebook. The preliminary allocation of meaning units to the main categories was iteratively and repeatedly discussed between all the authors until consensus was achieved. The content in each main category was collated by the first author (MN) and inductively abstracted into subcategories, with the conscious intention of preserving variation in the scope and depth of the data. Following this method, the researchers moved back and forth through the steps in order to validate, revise, and refine the findings. Although data saturation was indicated after the 19^th^ interview (new data did not add to the variation and scope of codes), all interviews were coded. Quotes from the data were chosen to illustrate and add depth to the descriptive text in each category and translated verbatim.

## Findings

The results show that the study participants have high expectations regarding the relative advantages of AI for the organisation, for health professionals, and for patients. They perceived the innovation source of AI to be located primarily in the organisation itself, but at the same time highlighted the need for outside professional assistance with the innovation design. Concerns were expressed around the evidence base of AI innovation; mainly for reasons of data security and undetected bias in the technology. They perceived that AI was highly complex—something reflected in the scope and nature of the technology itself, and in the steps involved in safely processing data and effectively managing clinical change. Perceptions of innovation trialability, adaptability, and costs were tentative because of the early stage of AI implementation in the organisation (Table 2).

**Table 2:** Main categories based on the constructs in the Innovation domain in the Consolidated Framework for Implementation Research, with subcategories and illustrative quotes

Main categories and subcategories	Illustrative quotes
Innovation relative advantage	
• Decision support for managers/leaders • Decision support for healthcare professionals • Better health outcomes for patients • Early detection of disease • Social impetus	“…understand our activities in a better way, so that you can make wise decisions… In part you can understand the medical development… and you can get a better sense of financial connections and relationships… when you can build together the activities we conduct… a bit tighter than what we have been able to do before” (2)
	“I want to know if it’s a clot somewhere or if it’s tied to some form of cancer, those are the answers I want as a treating physician… I think that an AI solution might become a form of support for decision-making” (1)
	“From a patient perspective… I think mostly… perhaps things like quality, well for the patient, both that it improves but also that we get faster, quicker assessments” (9)
	“That’s what I envision with AI. In the clinical work, AI has an amazing ability to assemble a large amount of information and see patterns in it” (10)
	“We are currently together with the university and region, tying up the big sharks to get them to join us and finance and develop things but also to get more companies from (our region, authors comment) This an industry for the future, … this is it” (8)
Innovation source	
• Development of AI internally through local strategic collaborations • Limited quality and safety awareness in smaller tech companies • Difficulties with external networking around AI	“We have all of the data in place, we have this system development department that can build things, we have a lot of knowledge in this house and a brave region and we are looking ahead and … we have the opportunity to prioritise things, …so I think it’s fully possible to build at the present time” (5)
	“Certain parts of the Medical Technology industry aren't used to critical thinking and scientific models, which is a requirement in healthcare… It’s the work that’s the most important part and the tricky part. I guess that’s the most time-consuming part too. A lot of Medical Technology companies also feel extremely frustrated about this. Because a lot of them feel that they have finished solutions. “You just have to get started, look you can save money or save lives” or whatever. Yeah great, and then I go over it, because I've made my own little check-list of things to investigate. Have you thought about this and that? Okay, what did you do when you were validating?” (2)
	“Maybe we shouldn’t talk about dangers or difficulties, but naturally we’re facing a challenge with current legislation being what it is. We’re noticing that even at this early stage. We’re being extremely cautious when it comes to selecting data. We have a great deal of respect for anything that’s individual. We can’t pick out just anything, in short, that’s how it is. That’s how it is and nor can we just pick out anything when it comes to private healthcare providers and compare and so on. We have a great legislation to adapt to as well and I think legislators need to review that as well and adapt” (6)
	“All regions are currently doing a great job just to achieve structure, that you can view things in a sensible way. It’s going to be a lot simpler in the future too, because you will maintain standards in an entirely different way. The result of this is that what I do, analysis and other things, becomes much easier. And then we have the standards that one will hopefully stick to “(10)
Innovation evidence-base	
• Uncertainty around opaque evidence-base • New understanding of evidence • Risks of biases feeding into the technology	“You can currently go down to the library and start digging through research reports. It takes a few hours, weeks, but you still work your own way towards that understanding, so to speak. And then with that amount… we’re still not going to find all of the research reports in this area of course, but I still feel that I can stand behind this. I’ve studied this, I trust this, it’s my assessment. I will also be taking responsibility if it doesn’t turn out that great” (7)
	“Then it’s also a bit unclear to me… where you can find science and proven experience… how is AI going to affect knowledge management? That parallel, I don’t quite understand it yet, because we are after all working on an evidence basis so it’s not like just ‘well I think we should do this because it seems…’ I mean, we don’t normally work like that in Swedish healthcare, when we know that we can produce evidence, and I’m still having a bit of a hard time seeing how those things will affect each other… we’re very used to having a lot of things to back up our decision and I think we can stay there.” (9)
	“There are so many things that can go wrong, if you look at AI specifically. Most things that are digital are thereby copyable. So along with the implementation of it, both advantages and disadvantages or risks are amplified. So if you have a built-in error, there consequence becomes massive. Since it is used in to such a high frequency. Those things are incredibly important to build a vaccination against, in approving process and things like that.” (2)
Innovation adaptability	
• AI will fit more naturally in some clinical contexts than in others	“I think that the conditions are very different… a general answer becomes way too hard… I think there’s a very good chance in diagnostics to achieve relatively fast implementation of AI, for example to help examine X-ray images, CTs or MRIs, where you do a fluoroscopy of soft parts in the body, it’s not as sensitive in terms of an individual’s privacy since you don’t know who is who. A lung looks like a lung and you can’t know who the person is by looking at the lung” (1)
Innovation trialability	
• Uncertainty about where to test AI in the organisation	“I think is going to need two organisations, just like we have now because they vary so much in nature. This data storage progress… needs to be very quality assured, it has to be very secure, it’ll be running every night, we don’t want disturbances in the system. This green section is a bit more exploratory… they’re a little bit different because… what’s important is to quickly be able to switch sources, switch ideas, switch development method… it’s a lot more exploratory” (5)
Innovation design	
• Need for external expertise to design the AI applications • Healthcare professionals currently have little knowledge about AI	“There needs to be some form of product from it in order for us to be able to use it. I think we have a challenge there, I would say, to create a product from this it probably has to be a company… it won’t work at all to put a click view in the hands of a clinician” (7)
	“One doctor out of all of the doctors I have met… wanted the routines printed out and placed on his desk. One single doctor out of all the ones I met with. Everyone else wanted it digitally and to be able to access it quickly and easily… it can’t be too complicated and it can be time consuming, because then it will lead to nothing” (4)
	“You kind of get the sense of the “beautiful new world”, something along the lines of that, but in reality it’s actually just a decent, advanced statistic and probability theory” (3)
Innovation complexity	
• Uncertainty about what AI is and is not • Lack of guidance for decisions about AI deployment in the organisation • Expectations of change resistance • Expectations of AI-scepticism and lack of trust	“In principle you have to press a button and you generate an answer and they can’t malfunction, but somewhere you still need to have a explanatory background involving the complexity. You need to very honest about that: these are the parameters that primarily form the basis of these AI decisions. If you haven’t included all of the parts you can’t mention them. Certain parts may not even be possible to add” (1)
	“You also understand what it entails to have an organised insertion, the procurement department, system management, knowledge department, digitalisation department… and then of course economy and communication, you know they are really complex systems… sometimes I’m a bit concerned about people who really don’t get it. These are really important discussions that need to be held with administration management so that you have a consensus. If you’re going to invest in areas where you know that you’re making very subjective assessments or where you’re where you have really high flows?… I mean we normally have really high flows in some cases, or do you invest where you have very small flows? There is an infinite number of perspectives, I just think that when it comes to issues like this it’s very important to think carefully so that you can motivate your reasoning” (9)
	“It’s unavoidable… that the every day routine for our employees will change …One of the even bigger challenges in addition to us needing to readjust is that we’re going to stop doing things. You’re going to stop doing things because they’re not creating value, and instead you’re going to do this. Here it’s not about the resistance to these services or these technical solutions, it’s that we want to continue with the old” (14)
	“If the business hasn’t said that there is a need and you say that this will improve things, there’s not a lot of motivation and benefit there I think” (1)
	“I think that it’s that you trust so much in yourself in your profession, occupational role, that I think you have a hard time allowing some other type of machine or data or something else to make that assessment somehow, you want to… you don’t quite trust it” (12)
	“If you’re going to build trust you need to know that what you’re working with actually provides you with that” (4)
	“The trust issue is important and most especially that you would be giving, make errors. What we talked about, that you get locked too soon. You miss something, miss something serious. At least when you’re working with health and healthcare that’s the most serious bit, I would say. If it happens we lose trust right away. That’s enough, and the issue of responsibility is a really difficult one. So I go on what Doctor AI recommended, and where is the burden of proof? Maybe I took a quick peek at these suggestions and felt that it sounded pretty good as soon on. I chose to use them, but can I blame Doctor AI?” (1)
Innovation cost	
• No state-allocated resources for implementation and roll-out of AI • Uncertainty about the level of costs involved in the future larger-scale implementation of AI	“You need to be able to allocate resources, time. You also need to finance it. We often forget that and think that we can manage it with the existing budget, but no you can’t. You need to allocate resources and money in order to succeed” (04)
	“Here it’s about prioritising the things that will benefit us the most, in some way. And then of course at the same time we need to have a high degree of development. We need to have some wise decision-makers here that can take part and still make some oriented decisions on what you need. Because it’s not cheap either, I can’t. It’s going to require a fair amount of time and quite a lot of development and things like that” (6)
	“AI will come and will most likely be a degree of priority in politics and for higher officials. The consequence will probably be that we won’t do it all. It’s quite likely that there will actually be… consequences for other things that also need to be done and followed up on… somewhere you’re going to need to make the cut too. They will become tough priority decisions to make, but somewhere we still have a line structure, we have politics, higher officials, where [the decisions] need to be made, and they become a matter of priority, where the resources will be spent. I’m doubtful that we’re going to get more resources” (7)

### Innovation relative advantage

Participants believed that a relative advantage of AI as an innovation to be implemented in healthcare lies in its effective and comprehensive management of large volumes of data from different sources, and in particular, data from the data warehouse of the organisation itself. Participants saw AI as part of a necessary development, as healthcare would not, in the long run, be able to keep up with the population’s healthcare needs. The application of AI technology was thought to enable decision-makers to allocate resources where they are most needed within the organisation. Management decisions regarding organisational changes in primary care units and hospitals were considered to be supported through the aggregation of data on outcomes from various care activities at multiple sites.

The participants also perceived AI’s potential to support professional decision-making in clinical care. AI was specifically perceived to be able to contribute through its ability to analyse images from digital imaging systems with a high level of precision and time effectiveness. The capacity of AI was perceived to be superior to the human analysing ability of even very clinically experienced professionals, in that it was not only more efficient and precise but also less biased.

AI was not perceived as replacing the need for human interaction between caregivers and patients, as this would provide other information, such as the patient’s preferences and state of mind. On the other hand, participants thought that AI could be equivalent to a colleague as a ‘second opinion’ in situations of uncertainty.

The participants described that AI could serve as a warning or a ‘yellow flag’, alerting healthcare professionals to clinical data that needs to be taken into account given a certain situation and specific conditions. The participants considered this to be uncontroversial, as they saw AI as just another tool to help healthcare professionals in their clinical work.

The participants had great expectations for possible AI-based applications that could about in the future. ‘Digital triage’ was perceived as an attractive idea to help empower patients in their own care and to achieve more effective care; acting as a self-help tool for some patients. This was expected to generate more time for clinicians to spend working with vulnerable patients. They also envisaged that standard health information could be collected from the patient and an AI-informed selection of laboratory tests could be completed prior to the primary healthcare visit, making the patient–provider encounter more informed and time-efficient.

Another aspect of perceived relative advantage was the potential of AI for discovering previously unknown patterns of care flow and its early detection of disease, facilitating health predictions for individual patients or groups of at-risk patients. Participants highlighted the AI algorithm’s ability to impartially discern clinical patterns based on multiple data sources without the need for prior clinical training, preunderstandings, or assumptions.

### Innovation source

Regarding the innovation source subdomain, participants thought that AI would primarily be internally developed in the near future. They thought that their organisation had some readiness to develop AI because of a relatively long history of investing in AI. Strategic leadership in the county council was perceived to have supported AI development and research early on, which led to a perception of local ownership in AI development within the county council.

AI as an innovation was perceived as a ‘hot topic’, and collaborations with universities and other actors like those in industry were seen as strategic, allowing the county council to take advantage of this ‘window of opportunity’. Some even expected local healthcare professionals to actively participate in the development of AI-based applications for use in their own field of interest.

Networking around the use of AI within the larger national healthcare system was perceived as a slower and more cumbersome process than regional collaboration with specific tech companies. However, participants believed that many tech companies were not equipped to follow the accepted quality and safety standards in healthcare, which led to hesitations about relying on these companies for AI implementation.

### Innovation evidence base

Although the participants described the innovation’s evidence base to be of key importance in AI implementation, they perceived the evidence base itself to be highly questionable. The participants felt they lacked control over the long chains of data processing and perceived that they had no insight into which process decisions were made along the way, for which reasons, and by whom.

Some participants perceived that it was possible to take the positive results of using AI as proof of its effectiveness, but highlighted the risk of bias in the data upon which the technology is based and how health data were processed. They felt strongly about the need for quality and safety control of AI-based applications; considering the consequences of faulty and skewed data-processing in AI and the large potential impact AI imperfections could have on managerial and health outcomes.

The participants thought that due to data being transferred between systems, aggregated, and repackaged, the original data would become increasingly difficult to retrieve and use.

The participants perceived that the mathematical complexity of AI prohibited an easy understanding of the reasoning that lay behind the information presented by AI; they characterised AI as a ‘little black box’. One of the reasons for questioning the evidence base of AI-based applications was that the knowledge-base and data behind AI could not be verified in traditional and transparent ways, like reading up on relevant scientific research findings.

### Innovation adaptability

The participants perceived AI to have a degree of adaptability, but they also believed it to fit more naturally into some clinical contexts than in others. Care units relying on medical imaging techniques as an important work tool were perceived as especially prepared to make changes toward AI-based diagnostic support. Areas that were highlighted in this aspect were radiology, pathology, clinical laboratory medicine, and dermatology. The participants thought that using AI would encounter barriers in other work units because of the perceived need to protect sensitive personal data.

### Innovation trialability

Regarding the innovation’s trialability, participants suggested testing AI on a small scale in the organisation but did not discuss any ways to retract the implementation, if necessary. The current use of AI for managing care within the organisation was experienced as gaining in importance and was tested in an ongoing process. The participants expected to be able to test a small number of AI-based applications in clinical contexts within the next few years; partly due to having observed that IT systems had prepared the technical infrastructure for AI ‘behind the scenes’.

AI-based digital imaging tools intended for use as diagnostic tools were seen as feasible for testing in clinical use, but participants also perceived it would be necessary to create more opportunities for testing other AI algorithms developed for use in care processes in clinical practice. The participants tentatively discussed where new AI-based applications could be appropriate and feasible; e.g. in situations that are a step removed from patient–provider encounters. They also reasoned about the usefulness of AI in situations of broader diagnostic uncertainty; in primary care consultations, for example. Some participants perceived that AI is already more or less informally present in some clinical contexts.

### Innovation design

Participants perceived that AI’s innovation design will be important for future implementation. They imagined that AI applications in healthcare need the AI component to be as simple to use as possible, while at the same time should be designed to target complex problems healthcare professionals need help in solving.

Participants were not convinced that the healthcare organisation itself could manage to design the AI applications without external expertise. They perceived that only developing AI models and algorithms were insufficient when it came to integrating the technology into practice and that the technical functions of AI needed to be integrated into user-friendly products designed for the use of healthcare professionals. They also perceived that future IT infrastructure development was necessary to integrate AI into IT systems for ease of use. Participants believed that AI could have different designs based on the same data but tailored to users of different professional backgrounds and patients with differing levels of digital and health literacy.

### Innovation complexity

Participants perceived multifaceted innovation complexity in the implementation and use of AI. They believed that there are many competing and occasionally conflicting opinions about what AI is and what it is not. Decisions about investments in AI were the remit of top-level management, but participants expressed a lack of guidance pertaining to decisions connected to AI. They wanted their decisions to be based on a thorough knowledge of the AI technology itself, and which type of problems it can be expected to solve. However, they were unclear as to which criteria should be used for decision-making about how and where to start applying AI in clinical practice.

The participants highlighted that collecting large volumes of data was not realistic at present, as health data were fragmented in the system, and current IT systems were not mutually compatible. Sharing data and exchanging knowledge between county councils was anticipated to be difficult, as different county councils make independent choices concerning how to build the data warehouses and which technologies and suppliers to invest in. The participants perceived risks of privacy violation during managing, monitoring, and storing large volumes of sensitive health data from many different data sources, involving different IT systems and numerous staff in technical and medical capacities and storage in commercial facilities. They also said that current legislation prohibits data sharing between different caregiving agencies in county councils and municipalities.

Participants experienced that processes of change tend to move very slowly in healthcare. In addition to professional change resistance and organisational barriers, a high level of scepticism around AI was to be expected.

Participants also expected AI to be potentially challenging to professionals in their professional role, in situations where AI might possibly provide healthcare professionals and patients with information that was not previously available, and which they are currently unequipped and unprepared to deal with.

Many other factors were expected to add to the complexity of implementing AI: levels of digital literacy on the part of patients and healthcare professionals, varying interest levels in AI in different professional fields, levels of technological know-how available in the organisation, and the capacity of healthcare leadership at all levels.

Not being fully cognisant of the scope and depth of knowledge in AI was thought to have consequences for patient safety in clinical practice. The participants perceived that there were risks of staff becoming overly reliant on the knowledge provided by AI, which could lead to more limited use of clinical reasoning. They highlighted that repeatedly exercising professional judgement was necessary for developing clinical expertise over time. This was seen as especially important for younger professionals, but even more experienced clinicians could risk becoming overly confident in AI-informed decision support.

The participants expected that the issue of AI leading to transformative changes for healthcare professionals and patients needed to be discussed by society, as healthcare would change profoundly towards being more prevention-focused in the future, with citizens expected to be more in charge of managing their own health.

### Innovation costs

The participants could not estimate the innovation costs of AI technology at present, but perceived that no state-allocated resources were available for more large-scale implementation and roll-out of AI. The participants had varying perceptions as to the success of the organisation’s efforts in developing AI so far, with some applauding current and past AI development efforts, while others instead questioned the outcome of the resources that the organisation had used for AI development.

Participants were uncertain about the level of costs involved in the future larger-scale implementation of AI but feared that some currently ongoing research and development projects could suffer. Although purchasing AI products and upgrading IT system capacity was thought to be costly, some participants thought that IT infrastructure was ready and able to accommodate AI, which would alleviate costs over the long term. The cost of product development by external companies was perceived as a barrier to implementation in the short term, as procurement procedures at present do not apply to AI. Over a longer perspective, participants expected that the organisation could incur financial costs for purchase, support, and maintenance of AI technology. Future projections of costs was perceived to include the potential recruitment of AI-competent staff.

## Discussion

The study explored the perceptions of leaders in healthcare concerning the innovation characteristics of AI of relevance for the adoption, implementation and use of AI in their organisation. Their perceptions were categorised in accordance with the eight constructs in the Innovation domain in CFIR. The results show that participants had high expectations of the *innovation relative advantage* of AI even though they were not convinced of the *innovation evidence base of AI.* The leaders were more tentative in their perceptions of *innovation trialability*, *innovation adaptability*, *innovation design*, and *innovation costs*, because of the relatively early stage of deployment of AI in their organisation. Participants’ expressions concerning *innovation source* and *innovation complexity* were indicative of conflicting perceptions: there were both perceptions that AI could be developed internally *and* that it needed a commercial expertise to make it work, and participants expressed both that AI was simple *and* very complex to implement.

The degree to which the characteristics in the innovation domain were perceived by leaders to support their expected implementation of AI may be seen to reflect both technology-related aspects and socio-organisational (people-process) aspects of AI. Considerations related to the technology-related aspects of AI were mainly found in leaders’ perceptions related to navigating the complicated issues concerning weighing the ins and outs of data management and data security and limited knowledge of the evidence of AI against the promises of its advantage over the effectiveness of current healthcare. AI-relevant legislation protecting health data, limited technological standardisation, ethical issues and supporting legal infrastructure for data-sharing are barriers to tackle if the ambitious Swedish eHealth goals are to be realised [29]. The complexity around legal and logistical barriers for implementing AI in healthcare non-withstanding, the spread of AI in all parts of society was not doubted by the leaders in our study. It is notable that the current societal and political enthusiasm for the benefits of AI was prominent in all the interviews, and in some cases compensated some leaders’ more negative perceptions about its evidence base, even though the opacity in AI was highlighted. The lack of intuitive understanding of the theory underlying AI model development and the high level of mathematical and statistical complexity has been termed as the “black box problem” [30]. Similarly, the ways in which AI models achieve their results are not always comprehensible [31]. Among the most important weaknesses in AI are potential biases embedded both within algorithms and within the data used to train algorithms [32]. Some potential trialability of AI was perceived by the leaders in the study, mainly because of the infrastructure they had in the organisation for some to test a small number of AI-based applications. Research confirms that a necessary foundation for delivering AI in healthcare is the extensive use of electronic health records and a high degree of interoperability between IT systems, even though the latter is a problem in many instances [8].

Considerations related to the socio-organisational aspects in many cases concerned dealing with uncertainties. This was expressed as the conceptual ambiguity of AI and the expected difficulties of managing distrust of AI in parts of the organisation, the uncertainty around where to develop and test AI, which types of AI applications to prioritise, and managing an unknown budget for AI implementation. The leaders described complexity in relation to AI’s conceptual ambiguity, which they perceived as potentially detrimental to communication around the technology. The general understanding of AI is diffuse for several reasons. Firstly, the term AI is used in many different ways in computer science, engineering and healthcare [33]. Secondly, the characteristics of AI are continually evolving, and different types of “AI” exist in parallel [30, 31, 34]. Thus, how we use the term AI needs to be clarified to identify nuances and differences of the AI technologies and AI systems, and study the specific challenges involved in their safe and effective implementation [31]. Leaders’ expressions concerning the innovation source were indicative of conflicting perceptions: there were both perceptions that AI could and should be developed internally and that the organisation needed commercial expertise to make it work. Internally developed AI was seen as providing a real possibility of input by users (such as healthcare professionals and patients) on the innovation design, which could impact implementation at all levels. Indeed, research confirms that there is a need to incorporate expertise and knowledge from different user groups in the development of AI-based applications. Combining the expertise of both computer scientists and healthcare professionals is key for making meaningful use of the data [2]. Furthermore, collaboration between levels and parts of systems and between organisations is needed to counter the disruptiveness in care flows [35]. Regarding costs, the leaders expressed that many aspects are unclear about the trade-off between costs and profits. Research confirms that there is a need to describe the initial investment and operational costs for infrastructure and service delivery of AI in future studies and that other options to achieve a similar impact must be benchmarked to inform strategic planning [36].

The considerations that managers and leaders express around both technical aspects and socio-organisational aspects of AI as a technology to be implemented into their organisation are of interest because of their central importance in the implementation process. The importance of leadership is evident in several of the models and frameworks used in implementation science. However, while implementation science has increasingly drawn attention to the role of leadership for successful implementation of interventions and innovations in healthcare, there is relatively limited research on leadership in healthcare [13, 37–39] and especially in the field of AI implementation in healthcare. Leadership is usually defined in terms of a process of influence to achieve certain goals, i.e. guiding a group to accomplish a task [40]. The vast majority of the 28 participants in our study were leaders with a position as a manager in the county council and all were considered to be leaders with regard to the implementation of AI in healthcare. However, there may also be other persons in healthcare who are not necessarily formal managers but still have a great deal of influence on AI implementation. Physicians are often described as leaders in many healthcare contexts and their involvement in various implementation and improvement initiatives is often crucial for achieving desired goals [41]. Research has documented ambivalence among physicians towards AI, with diverse and sometimes contradictory viewpoints [42–44]. Concern has been expressed about a loss of autonomy among physicians and the increasing integration of AI into human-centred care, which some fear could lead to a gradual replacement of physicians with AI applications. Physicians gather and process medical information to make a diagnosis, which are tasks that could potentially be performed by AI applications [20, 45, 46]. In our study the leaders’ expressed expectations of mistrust among healthcare professions in health care. Research has documented a diversity in attitudes regarding AI among various stakeholders [44, 47, 48]. Thus, understanding what drives different stakeholders’ perceptions of AI in healthcare is important. Further research into how and why differing views are held could assist the development of strategies that accommodate such diversity of views.

## Study limitations and methodological considerations

The study has some limitations that need to be considered when interpreting the results. Participation in the study was voluntary, which means that the leaders who agreed to participate may have been particularly interested in the topic or may have vested interest in ongoing efforts to implement AI. However, it is difficult to determine how this might have affected the results. The innovation itself (i.e., AI) appeared to be interpreted by participants in different ways. The different terms that the participants used reflect the general immaturity of the language describing different forms of AI and the topic’s relative novelty for the participants. The manifold terms used when talking about AI are also indicative of the participants’ perception of AI as a “general purpose technology”. [33]. Future studies could benefit from a clearer delineation up-front of the different applications of the technology to understand more in-depth how each application is perceived by leaders in healthcare.

We applied CFIR in the analysis to categorise the findings concerning perceptions of AI. CFIR is a widely-used determinant framework to evaluate the implementation of interventions in healthcare. Determinant frameworks are used in implementation science to describe different types of influences on implementation that are hypothesised to—or have been found to—impact implementation outcomes. CFIR has previously been used to assess the implementation of AI applications in various settings [49, 50]. In this study, we limited its use to eight characteristics in the innovation domain in the CFIR framework [9] which we found suitable to address aspects of perceptions deemed relevant to the study aim.

The transferability of the study’s results is primarily limited to Swedish healthcare. However, key characteristics of AI may be generalisable beyond the Swedish context to inform stakeholders about possible facilitators and barriers for the adoption, implementation, and use of AI [51]. Communication around these determinants in a standardised manner may facilitate dialogue [52]. It should be remembered, however, that the characteristics of an innovation are not stable features, nor are they the only determinant of adoption and implementation. The interaction between the innovation, the intended adopters, and the context influences the adoption and implementation of innovations [53, 54].

The multidisciplinary research team was a strength of the study because it permitted different perspectives on the issue of leaders’ perceptions of AI. The team consisted of researchers with expertise in implementation science, intervention science, and health science with a specific focus on the implementation of health innovations (such as AI applications) in healthcare. Another strength was the relatively high number of interviews (*n* = 28) which added trustworthiness to the findings.

## Conclusions

In conclusion, this study found that Swedish healthcare leaders preparing to implement AI acknowledged the potential for AI to contribute to improvements in healthcare. They had high expectations of the *relative advantage* of AI but were less convinced of its *evidence base* (AI’s safety and effectiveness) and tentative in their perceptions of the innovation’s *trialability*,* adaptability*, *design*, and *costs.* This may have implications for future implementation of AI: to ensure the adoption, implementation, and sustained use of AI in healthcare, implementation strategies will likely need to be designed to manage perceptions of innovation characteristics of AI at the leadership level at early stages of the technology’s implementation. More in-depth knowledge is needed about perceptions of the barriers and facilitators to AI implementation in other stakeholder groups and about outcomes from the implementation of AI in real-world situations to develop strategies to support its implementation in practice.

## Supplementary Information

**Additional file 1.** Consolidated criteria for reporting qualitative studies (COREQ): 32-item checklist.

**Additional file 2.** Interview guide.

## Data Availability

Empirical material generated and/or analysed during the current study are not publicly available, but are available from the corresponding author on reasonable request.
