# Supplementary material for: Innovation in healthcare: leadership perceptions about the innovation characteristics of artificial intelligence—a qualitative interview study with healthcare leaders in Sweden
Source: Implement Sci Commun. 2023 Jul 18;4:81. doi: 10.1186/s43058-023-00458-8 (PMC10354990; doi:10.1186/s43058-023-00458-8)
Supplement: Supplementary file 2 — Additional file 2. Interview guide. [file 43058_2023_458_MOESM2_ESM.docx]

Additional File 2. Innovation in Healthcare: Healthcare leaders' perceptions about the innovation characteristics of artificial intelligence—A qualitative study in Sweden

**Interview guide**

**Introduction**

- This Region has assembled its health data in a platform to support quality development
- Funding was received in 2020 to test-case the introduction of AI
- The aim of this interview is to explore the different perspectives relating to the application in practice of these technologies
- The university employees conducting the interviews will start with recruiting participants at high levels in the healthcare organization
- Participation in the interview is voluntary and can be stopped at any time. The interview will be recorded. The research group will treat the recorded and transcribed data with the utmost confidentiality.

**Background questions**

1. What is your role and what are your duties and responsibilities?
2. Do you have any experience of implementing new technology in the healthcare organization? If you do, did the implementation influence professional roles or work flows?
3. What is your insight into the Region´s information-driven care initiative and the platform for healthdata analysis and research?
4. What are your own thoughts about using AI to improve healthcare? Any experience?
5. Which are the possibilities and opportunities to use AI in your part of the organization as you see it, from your perspective and with your knowledge about the technology?

**What is the problem or challenge and how do you perceive the implementation of AI in relation to that problem or challenge?**

1. How would you characterize your awareness, knowledge and/or earlier engagement in introducing AI-based solutions?
2. What is your perception of the challenges involved in the introduction of AI-based solutions?
3. Could you describe any areas of care that would have an added potential for care improvement?
4. Which type of improvement might one expect in these areas of care?
   1. Potential results?
   2. Influence on patients/population?
   3. Influence on employees?

**Implementing information-driven care using AI**

1. What do you think AI will primarily achhieve in practice?
   1. In which ways might AI influence staff´s current practice?
   2. How will it fit with the ways care is provided and organized for patients today? What would need to change?
   3. Which professional groups will be impacted and why?
2. How will AI influence your own professional role?
   1. Which resources and time will be needed from you in the future?
3. What unavoidable or unexpected consequences do you expect from introducing AI?
   1. Which barriers do you perceive of?
   2. Which risks do you perceive of?
4. How would you characterize successful implementation of AI?
5. What resources and/or time do you think will be needed to implement AI? Are there any allocated resoursec and/or time for facilitating AI implementation?

**Wrapping up the interview**

1. Is there anything else that feels important and that we have not covered in our conversation?
2. Is there anyone else you know that we should talk to about this issue?
3. In which way would you like to be involved in the exploration of the implementation of AI in the future?
